# Postoperative fasting is associated with longer ICU stay in oncologic patients undergoing elective surgery

**DOI:** 10.1186/s13741-022-00261-4

**Published:** 2022-08-02

**Authors:** Caroline Fachini, Claudio Z. Alan, Luciana V. Viana

**Affiliations:** 1 Critical Care Unit, Hospital Casa de Misericórdia, Rua Prof. Annes Dias, Porto Alegre, RS 295 Brazil; 2 Critical Care Unit, Hospital Mãe de Deus, Rua José de Alencar, Porto Alegre, RS 286 Brazil; 3 Endocrine Division and Medical Nutrition Division Hospital das Clínicas de Porto Alegre. Postgraduate Program: Endocrinology, Universidade Federal do Rio Grande do Sul, Porto Alegre, Brazil

**Keywords:** Fasting, Surgical oncology, Length of stay

## Abstract

**Background:**

Cancer patients present nutritional and complications risks during the postoperative period. Fasting contributes to surgical catabolic damage. This study evaluates the consequence of fasting time on the surgical outcomes of cancer patients undergoing elective surgeries.

**Methods:**

Prospective cohort, evaluating two categories of patients according to postoperative fasting: less than or greater than 24 h. Outcomes: Hospitalization time, 28-day mortality, ICU stay and infection rates.

**Discussion:**

We included 109 patients (57% men, 60 ± 15 years, BMI: 26 ± 5 kg/m^2^, SAPS3 43 ± 12), hepatectomy was the most frequent surgery (13.8%), and colon and rectum were the most common neoplasia (18.3%). The ICU stay was longer in postoperative fasting > 24 h (5.5 [4–8.25] vs. 3 [2–5] days, *p* < 0.001). Fasting > 24 h persisted as a risk factor for longer length of stay (LOS) in the ICU after adjustments. There were no differences in the mortality analysis within 28 days and total hospitalization time between groups. A tendency to experience more infections was observed in patients who fasted > 24 h (34.8% vs. 16.3%, *p* = 0.057). Onset of diet after the first 24 h postoperatively was a risk factor for longer ICU stay in cancer patients who underwent major surgeries.

**Graphical Abstract:**

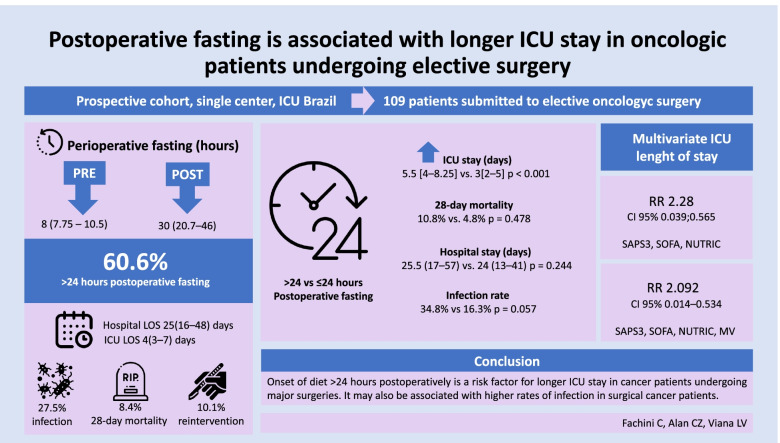

**Supplementary Information:**

The online version contains supplementary material available at 10.1186/s13741-022-00261-4.

## Background

Traditionally, perioperative fasting consists of *Nil per ol* (NPO) from midnight of the day before surgery until recovery of intestinal function in the postoperative period (Maltby 2006). This outdated practice persists despite new evidence that excessive fasting results in negative outcomes and delayed recovery after surgery (Shida et al. 2017).

Nutritional status is a strong independent predictor of poor postoperative outcomes (Weimann et al. 2017). Malnourished surgical patients have higher mortality rates, morbidity, hospital length of stay (LOS), readmission rates, and hospital costs (Correia and Waitzberg 2003; Wischmeyer et al. 2018; Williams et al. 2019). Therefore, perioperative nutritional therapy has been useful in improving surgical outcomes in these patients (Wischmeyer et al. 2018; Sandrucci et al. 2018). There are many evidence-based protocols for rapid postoperative recovery (Weimann et al. 2017; De-Aguilar-Nascimento et al. 2017; Lambert and Carey 2016; Dickerson 2019) that endorse the reduction of perioperative fasting, but the implementation and adherence to them is still limited (Lambert and Carey 2016).

Moreover, most rapid recovery guidelines for the postoperative period do not assess the impact of an isolated measure, but rather the whole bundle, to improve surgical outcomes and discharge time. Also, patients undergoing gastrointestinal (GI) and oncological surgeries are of special interest, since this population has the greatest risk of malnutrition.

We hypothesized that postoperative fasting time may be an independent marker of outcomes in the postoperative period of oncological surgeries. Thus, we evaluated the impact of postoperative fasting time on hospital LOS, mortality, ICU LOS, and the occurrence of infectious complications in adult patients admitted in the ICU due to oncologic elective surgery.

## Methods

We conducted a prospective cohort study in adult cancer patients undergoing elective surgery admitted for at least 48 h in the ICU of Hospital Santa Rita, between April 2018 and September 2019. It is a reference hospital in cancer treatments, receiving patients from all over southern Brazil, with a 10-beds ICU.

Exclusion criteria were patients under 18 years of age, undergoing emergency surgery, with length of stay in the ICU < 48 h, imminent risk of death (within 24 h), pregnant women and patients with exclusive palliative care.

On the first day of ICU admission, data on age, gender, weight, comorbidities and severity scores APACHE II (Acute Physiology and Chronic Health II) (LeGall et al. 1986), SAPS3 (Simplified Acute Physiology Score 3) (Silva Jr et al. 2010), SOFA (Sequential Organ Failure Assessment) (Vincent et al. 1996), NUTRIC (Nutrition Risk in Critically Ill) (Heyland et al. 2011), and Charlson (Charlson et al. 1994) scores were collected from the patients included. Weight was measured with a portable scale in the ward or a bed scale during the first day in the ICU, height was measured with measuring tape or estimated with the Chumlea predictive formula (Mitchell and Lipschitz 1982), and both were used to estimate BMI (i.e., weight [kg]/height^2^ [m^2^]). Calf circumference was measured with an inelastic tape in the ICU bed when the patient arrived, preferably on the left calf. Use of vasopressors, mechanical ventilation, and the need for renal replacement therapy were recorded. All data were prospectively assessed from electronic medical records in the Tasy System and from the Sistema Epimed Monitor® (Zampieri et al. 2017), a cloud-based national registry for ICU patients.

Patients were classified as nourished or malnourished by criteria of at least one nutritional assessment scores: PG-SGA (Patient–Generated Subjective Global Assessment) or MNA (Mini Nutritional Assessment) (Silva Fink 2015; Baker et al. 1982). In the case of disagreement between the scores, the PG-SGA was used as a tiebreaker because it is the most validated scale in the literature for cancer patients (Instituto Nacional de Câncer José Alencar Gomes da Silva 2016). The NUTRIC tool was used to assess the nutritional risk of ICU patients; the cutoff point used was ≥ 5 and without the evaluation of interleukin-6, since it is not available in our service (Heyland et al. 2011).

All aspects related to surgical technique followed the usual routine of the service and the surgeon, such as the use of drains or probes, manual or staple sutures, open or minimally invasive surgery or colon preparation.

Fasting times and data on the duration of surgery were obtained from the anesthetic sheet and surgical report in the medical records. Preoperative and postoperative fasting time were extracted from the records. From this data, fasting times were calculated. Patients were stratified according to postoperative fasting time into two groups: ≤ 24 h or > 24 h. Any caloric intake by enteral or oral route was considered the end of fasting period. After onset of the diet, patients were followed for three days or until the moment of discharge from the ICU. Route of administration, type of diet, number of calories per kilogram of the patient and the 3-day caloric target were evaluated, and total caloric intake was considered adequate if it was between 20 and 25 kcal/kg on the third day (McClave et al. 2016). Diet tolerance (measured in the first, second and third day of diet attempt intake) was verified with clinical signs and symptoms, such as nausea, vomiting, and bloating. The acceptance of the oral diet was assessed with intake control performed by the nutritionist, and acceptance of enteral and parenteral nutrition was assessed based on infusion records. Glycemic control was performed according to the routine of the service and hyperglycemia was defined as capillary glucose > 180 mg/dl (Finfer et al. 2009). The variable norepinephrine use (categorical) was considered as “yes” when the dose of norepinephrine > 0.1 mcg/kg/min. Infection rate (at any site) was also evaluated.

For mortality outcome, patients were followed-up for 28 days after surgery. Patients discharged from the hospital before 28 postoperative days were assessed by telephone call.

### Statistical analysis

Considering a previous study showing prolonged hospital LOS in patients with longer fasting periods was present in approximately 47% of patients (Assis et al. 2014a), we estimated a sample size of 108 patients to assess an absolute difference around 27% on hospital LOS. Data were entered in an Excel program and later exported to the SPSS v.20.0 program for statistical analysis. Categorical variables were described by frequencies and percentages. The symmetry of the variables was verified with the Kolmogorov-Smirnov test. The quantitative variables with symmetric distribution were described by means and standard deviations and those with asymmetric distribution by medians and interquartile intervals.

Categorical variables were associated by the chi-square test. The quantitative variables with symmetric distribution were compared by Student’s *t* test for independent samples. The variables with asymmetric distribution were compared by the Mann-Whitney *U* test. A linear regression analysis was used to evaluate the relationship between factors associated with the outcome length of ICU stay. The significance level established for the comparisons was 5%. Nonparametric data were logged to meet the model’s premise. The dependent variables were chosen based on their significance in the univariate analysis or their biological relevance.

### Ethics

This study was conducted in accordance to the Declaration of Helsinki and was approved by the Ethics Committee of the Irmandade Santa Casa de Misericórdia de Porto Alegre and it was registered in the Plataforma Brasil (CAAE: 81019617.3.0000.5335). An informed consent form was obtained from all patients or relatives of the patients who participated in the study.

## Results

### Population overview

**Fig. 1:**
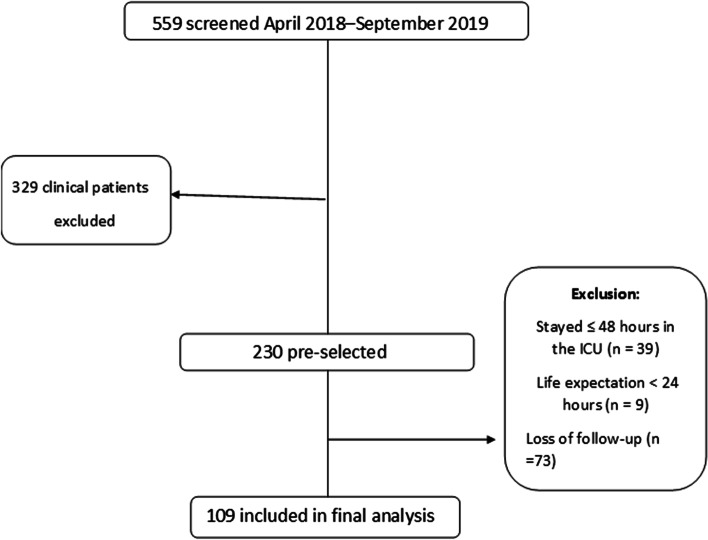
Flowchart of patients included in the study

During the study period, 559 patients were admitted to the ICU of Hospital Santa Rita, being 109 patients due to elective oncological surgery, as shown in Fig. 1. Most patients were men (57%), aged 60 ± 15 years, with BMI 26 ± 5 kg/m^2^. The mean SAPS3 score was 43 ± 12, as shown in Table 1. Hepatectomy was the most frequently performed surgery (13.8% of the cases), and colon and rectum neoplasia were the most frequent neoplasia, accounting for 18.3% of the operated patients. The prevalence of malnutrition was 38.3%. Hospital LOS was 25 (15.5–47.5) days, with 4 (3–7) days of ICU LOS; 28-day mortality was 8.4%, infection rate was 27.5% and surgical re-intervention was 10.1%.

**Table 1:** Patient characteristics according to postoperative fasting time

Patient characteristics	Overall *n* = 109	Fasting ≤ 24 h *n* = 43	Fasting > 24 h *n* = 66	*P* value
Age (years, mean ± standard deviation)	60.03 ± 15.2	58.81 ± 13.97	60.82 ± 16.09	0.478
Men (*n*; %)	57 (52.3)	25 (58.1)	32 (48.5)	0.429
Weight (kg; range)	73 (62–82.5)	73 (62–79)	73 (62–86)	0.91
BMI (kg/m^2^, mean ± standard deviation)	26.91 ± 5.7	26.76 ± 4.83	27.00 ± 6.2	0.831
SAPS3 (mean ± standard deviation)	43.6 ± 12.09	39.4 ± 9.42	46.48 ± 12.8	0.01
SOFA (*n*; range)	3 (1–6)	2 (1–4)	4 (1–7)	0.02
NUTRIC (*n*; range)	2 (2–4)	2 (1–3)	3 (2–4)	0.07
Malnutrition (*n*; %)	36 (38.3)	15 (39.51)	21 (37.5)	1.000
CC (cm; mean ± standard deviation)	35.6 ± 4.12	35.87 ± 3.4	35.55 ± 4.5	0.680
Charlson (*n*; range)	3 (2–6)	2 (2–6)	3 (2–6)	0.22
Ischemic heart disease (*n*; %)	11 (10.6)	5 (11.6)	6 (9.1)	0.75
Cardiac failure (*n*; %)	6 (5.5)	0 (0.0)	6 (9.1)	0.79
Diabetes (*n*; %)	21 (19.3)	8 (18.6)	13 (19.7)	1.00
Hypertension (*n*; %)	38 (34.9)	16 (37.2)	22 (33.3)	0.834
COPD (*n*; %)	9 (8.3)	2 (4.7)	7 (10.6)	0.478
Smoking (*n*; %)	26 (23.9)	11 (25.6)	15 (22.7)	0.911
Alcoholism (*n*; %)	8 (7.3)	3 (7.0)	5 (7.6)	1.00
Liver disease (*n*; %)	2 (1.8)	2 (4.7)	0 (0.0)	0.153
HIV (*n*; %)	2 (1.8)	1(2.3)	1 (1.5)	1.00
Other comorbidities (*n*; %)	39 (35.8)	16 (37.2)	23 (34.8)	0.963
Pre-fasting (h; range)	8 (7.7–10.5)	8 (8–13)	8 (7.5–9.62)	0.856
Post-fasting (h; range)	30 (20.7–46)	19.5 (15.5–22)	45 (31–63.12)	0.000
Total fasting (h; range)	42 (34.5–60.5)	34 (32–36)	58 (46–81)	0.000
Primary tumor (*n*; %)	71 (65.1)	28 (65.1)	43 (65.2)	1.00
Metastases (*n*; %)	57 (52.3)	21 (48.8)	36 (54.5)	0.69
Chemotherapy (*n*; %)	45 (41.3)	17 (39.5)	28 (42.4)	0.92
Radiotherapy (*n*; %)	21 (19.3)	5 (11.6)	16 (24.2)	0.166
Re-intervention (*n*; %)	11(10.1)	2 (4.7)	9 (13.6)	0.195
RRT (*n*; %)	6 (5.5)	1 (2.3)	5 (7.6)	0.4
Mechanical ventilation (*n*; %)	43 (39.4)	1 (23.3)	33 (50)	0.010
Noradrenaline (*n*; %)	28 (25.7)	9 (20.9)	19 (28.8)	0.000
Another vasopressor (*n*; %)	11 (10.1)	0 (0.0)	11 (16.7)	0.003

*Abbreviations*: *SAPS3* simplified acute physiology score 3, *SOFA* sequential organ failure assessment, *NUTRIC* nutrition risk in critically ill, *BMI* body mass index, *CC* calf circumference, *COPD* chronic obstructive pulmonary disease, *HIV* acquired immunodeficiency syndrome, *RRT* renal replacement therapy

### Nutritional characteristics

Patients with postoperative fasting > 24 h presented a nutritional risk (estimated by NUTRIC) higher than those with fasting time < 24 h (15.2% [10] vs. 2.3% [1], *p* = 0.047). There was no statistical difference in malnutrition prevalence between the two fasting groups (37.5% [21] vs. 39.5% [15], *p* = 0.99).

The preferred postoperative route of nutrition was the oral route (66.1%), followed by enteral nutrition (13.8%) and the parenteral route (8.3%). Of the 10 patients who received parenteral nutrition (PN) early in the postoperative period (before the fifth day), five underwent cytoreductive surgeries with hyperthermic intraoperative intraperitoneal chemotherapy (HIPEC) and three cytoreductive surgeries without HIPEC. Only 46.8% (51/109) of the patients reached the caloric target by the third day of ICU stay. We found a diet tolerance of 86% on the first day, followed by 84% and 82.4% on the second and third day, respectively. The most frequent symptom after the beginning of the diet was abdominal bloating, which reached 11.9% on the first day, while other signs of intolerance, such as vomiting or diarrhea, were present in 6.4% and 0.9%, respectively.

### Fasting time

Median preoperative fasting time was 8 (7.75–10.5) h and postoperative was 30 (20.7–46) h; in 39.4% of the patients postoperative fasting was ≤ 24 h. The ICU LOS was higher in the group that fasted for more than 24 h in the postoperative period (5.5 [4–8.25] vs. 3[2–5], *p* < 0.001), without significant difference for mortality within 28 days (10.8% vs. 4.8%, *p* = 0.478) or length of hospital stay (25.5 [17–57] vs. 24 [13–41], *p* = 0.244) (Supplemental Table 3). The proportion of patients with prolonged hospital LOS was not different when comparing patients that fasted > 24 h with the < 24 h group (65.2% vs 60.5%, *p* = 0.686). Our data suggest a higher infection rate was observed in patients who fasted for more than 24 h, although without statistical significance (34.8% vs 16.3%, *p* = 0.057).

In the multivariate analysis (Table 2), postoperative fasting > 24 h is an independent risk factor for longer ICU stay (in this logarithmic form) after correction for severity and nutritional risk score (model 1). A second model was elaborated (model 2), with inclusion of the variable mechanical ventilation and the statistical power of postoperative fasting longer than 24 h was maintained. In a sensitivity analysis, exclusion of patients who received parenteral nutrition did not alter the results (data not shown).

**Table 2:** Multivariate linear regression model-depended variable length of ICU* stay (log) for the inpatients included in the study

	*B*	*T*	CI	*P* value
First model				
Fasting > 24 h	0.208	2.28	0.039–0.565	0.025
SAPS3	0.341	3.282	0.008–0.032	0.001
SOFA	0.139	1.180	− 0.024–0.093	0.241
NUTRIC	− 0.091	− 0.797	− 0.147–0.063	0.427
Second model				
Fasting > 24 h	0.274	2.092	0.014–0.534	0.039
SAPS3	0.015	2.288	0.002–0.028	0.024
SOFA	0.02	0.683	-0.039–0.079	0.496
NUTRIC	− 0.032	− 0.609	− 0.136–(− 0.072)	0.544
MV ICU	− 0.318	− 0.218	− 0.612–(− 0.024)	0.034

*Abbreviations*: *ICU* intensive care unit, *SAPS3* simplified acute physiology score 3, *SOFA* sequential organ failure assessment, *NUTRIC* nutrition risk in critically ill, *MV* mechanical ventilation

Higher capillary glucose levels in patients fasting ≤ 24 h were found at first postoperative day. There was no difference between the groups, relative to hyperglycemia or administration of insulin at any other day (Supplemental Table 4).

## Discussion

This study was conducted to evaluate the impact of surgical fasting and length of hospital and ICU stay, mortality and infectious complications. In this prospective study, conducted exclusively with cancer patients, we showed that postoperative fasting time greater than 24 h is a risk factor for longer ICU stay, adjusting for severity of the patients according to SAPS3, SOFA, and NUTRIC scores. We also showed a trend to higher infection rates in the category of patients with > 24 h postoperative fasting. No difference in mortality was found between the two groups, but the mortality rate in our study was similar to that found in literature for the same population (Ghaferi et al. 2009; Finlayson et al. 2003).

The safety of reducing postoperative fasting time is already defined by international and national postoperative guidelines (Weimann et al. 2017; Lambert and Carey 2016; Khalid et al. 2010; Berger and Chiolero 2009; Reignier et al. 2018; Nelson et al. 2019; Arends et al. 2017). Analysis of surgical outcomes in these protocols are thought as a bundle that includes surgical preparation, the procedure itself and recovery in the postoperative period (van Zelm et al. 2020). Evaluating each aspect of care bundles individually is difficult. In a meta-analysis evaluated early versus late feeding in patients submitted to GI surgery, there was no reduction of mortality or LOS in the hospital. This findings are similar to similar to our study, but in the meta-analyses ICU stay was not analyzed (Osland et al. 2011). Also, in another study conducted in the south of Brazil, prolonged fasting was an independent risk factor both for infection and prolonged hospital stay (Assis et al. 2014b). However, this study was conducted in general surgery patients (ward and ICU), not only with oncologic patients. For gynecological surgery, systematic review shows benefits in early oral intake (liquid/foods): faster recovery of bowel function (Charoenkwan and Matovinovic 2014); however, this benefits are inconclusive in lower gastrointestinal surgery (Herbert et al. 2018). Also, early enteral feeding seems to reduce hospital LOS (Herbert et al. 2019).

There are no robust data in the literature evaluating the impact of fasting time on postoperative outcomes separately from care bundles, specifically for cancer patients. In our study, we focus in this specific population. Cancer patients differ from the general surgical population because they have a higher incidence of malnutrition, both due to the neoplasia itself and the cancer therapy (Arends et al. 2017), resulting in a higher incidence of postoperative complications. The prevalence of malnutrition in cancer patients ranges from 20% to more than 70% worldwide, and GI, head and neck, liver and lung cancers have the highest rates of malnutrition (Hébuterne et al. 2014; Silva et al. 2015). In our study, we found a prevalence of 38.3% of malnourished patients. In cancer treatment, surgical complications and prolonged hospital stay may delay the onset of postoperative chemotherapy, which in turn can reduce long-term survival (Ljungqvist et al. 2017), in addition to greatly increasing treatment-related costs (Vonlanthen et al. 2011).

Although the goal of implementing evidence-based approaches is the improvement in patient care, reduced ICU LOS drives to a decrease in hospital costs. Estimates in the literature of ICU stay daily costs differ considerably, according to the complexity of the health institution, location and case-mix, ranging from £706.69 ($1,000.00 USD)/day to £3533.45 ($5,000.00 USD)/day. Surgical admissions used to be cheaper than clinical ones (Gershengorn et al. 2015). In our study, we found a median reduction of ICU stays of two days. Sogayar et al. documented a cost of £660.05 ($934 USD) (interquartile range [IQR] 735–1170; 95% confidence interval [CI] 897–963) per day of ICU stay of septic patients, in a large multicenter study conducted in the Brazilian population (Sogayar et al. 2008), but we have no studies with the surgical population in Brazil.

Although the patient’s tolerance to restarting the diet is not universal, it is generally high (above 70%) (Gianotti et al. 2011; Dag et al. 2011). We had a tolerance rate greater than 80% for the diet during the postoperative period. Abdominal bloating was the most important complication on the first day, which is in accordance with international literature. It seems to be safe to start the diet early in the postoperative period (Ljungqvist et al. 2017; Vallejo et al. 2017); however, only 46.8% of our patients reached the calculated caloric target in three days after diet onset (McClave et al. 2016; Vallejo et al. 2017). Definition of caloric target and the time in which the target should be reached for critically ill patients remains controversial (Berger et al. 2017; Heidegger et al. 2013; Zusman et al. 2016), as well as its impact on ICU outcomes, ICU LOS, and mortality. We could not find differences on mortality, infection rates, ICU, and hospital LOS comparing patients who reached or did not reach the caloric target at 3 days. However, our study was not designed to assess this research question (data not shown).

### Limitations

Our study has limitations. The first limitation is its observational nature. Nutritional data should be carefully evaluated in the ICU population since many factors can influence the outcomes. Patients who fasted for more than 24 h were also the most severe cases (higher rates intensive support as mechanical ventilation and noradrenaline use, higher SAPS3, and SOFA scores). It could have delayed the onset of diet due to the misguided recommendation in the literature regarding the onset of diet in patients with hemodynamic instability (Khalid et al. 2010; Berger and Chiolero 2009; Reignier et al. 2018). Nonetheless, our results were maintained in the multivariate analysis, which included severity scores as confounders, even when a second model was performed including mechanical ventilation as a covariate. There are many unmeasured factors related to dietary intake in the postoperative status like surgeon’s preference and subjective evaluation of the physiology status of the patient. To overcome these barriers, both intensivists and nutritionist of our multidisciplinary team review diet charts of each patient and discuss the feeding protocols with the staff. Finally, this was a single-center study. The extrapolation of our results to other centers requires additional consideration regarding local practices and procedures.

## Conclusion

Onset of diet after 24 h postoperatively is a risk factor for longer ICU stay in cancer patients undergoing major surgeries. It may also be associated with higher rates of infection in surgical cancer patients. More studies might assess this data in different settings and its impact on other outcomes, with potential to impact on postoperative diet recommendations in surgical cancer patients.

## Supplementary Information

**Additional file 1: Supplemental Table 3.** Univariate analysis of the outcomes of interest of the patients included in the study. **Supplemental Table 4.** Glucose blood level and use of insulin during the first days of the study.

## Data Availability

The datasets analyzed during the current study are available from the corresponding author on reasonable request.

## References

[CR1] Arends J, Baracos V, Bertz H, Bozzetti F, Calder PC, Deutz NEP (2017). ESPEN expert group recommendations for action against cancer-related malnutrition. Clin Nutr..

[CR2] Assis MC, Silveira CR, Beghetto MG, Mello E (2014). Is duration of postoperative fasting associated with infection and prolonged length of stay in surgical patients?. Nutr Hosp..

[CR3] Assis MCS, Silveira CRM, Beghetto MG, Mello ED (2014). ¿Duracion del ayuno postoperatorio se asocia con la infeccion y la estancia prolongada en pacientes quirurgicos?. Nutr Hosp..

[CR4] Baker JP, Detsky AS, Wesson DE (1982). Nutritional assessment: a comparison of clinical judgment and objective measurements. N Engl J Med..

[CR5] Berger MM, Chiolero RL (2009). Enteral nutrition and cardiovascular failure: from myths to clinical practice. J Parenter Enteral Nutr..

[CR6] Berger MM, Pichard C, Fontaine E (2017). Optimal energy delivery and measured energy expenditure – impact of length of stay. Crit Care..

[CR7] Charlson M, Szatrowski TP, Peterson J, Gold J (1994). Validation of a combined comorbidity index. J Clin Epidemiol..

[CR8] Charoenkwan K, Matovinovic E. Early versus delayed oral fluids and food for reducing complications after major abdominal gynaecologic surgery. Cochrane Database Syst Rev*. *2014;2014:12. 10.1002/14651858.CD004508.pub4 PMC7044077 25502897

[CR9] Correia MI, Waitzberg DL (2003). The impact of malnutrition on morbidity, mortality, length of hospital stay and costs evaluated through a multivariate model analysis. Clin Nutr..

[CR10] Dag A, Colak T, Turkmenoglu O, Gundogdu R, Aydin S (2011). A randomized controlled trial evaluating early versus traditional oral feeding after colorectal surgery. Clinics (Sao Paulo)..

[CR11] De-Aguilar-Nascimento JE, Salomão AB, Waitzberg DL, Dock-Nascimento DB, Correa MITD, Campos ACL (2017). Diretriz ACERTO de intervenções nutricionais no perioperatório em cirurgia geral eletiva. Rev Col Bras Cir..

[CR12] Dickerson SC (2019). Perioperative guidelines in anesthesia. Otolaryngol Clin North Am..

[CR13] Finfer S, Chittock DR, Su SY (2009). Intensive versus conventional glucose control in critically Ill patients. N Engl J Med..

[CR14] Finlayson EVA, Goodney PP, Birkmeyer JD (2003). Hospital volume and operative mortality in cancer surgery a national study. Arch Surg..

[CR15] Gershengorn HB, Garland A, Gong MN (2015). Patterns of daily costs differ for medical and surgical intensive care unit patients. Ann Am Thorac Soc..

[CR16] Ghaferi AA, Birkmeyer JD, Dimick JB (2009). Variation in Hospital Mortality Associated with Inpatient Surgery. N. Engl. J. Med..

[CR17] Gianotti L, Nespoli L, Torselli L, Panelli M, Nespoli A (2011). Safety, feasibility, and tolerance of early oral feeding after colorectal resection outside an enhanced recovery after surgery (ERAS) program. Int J Colorectal Dis..

[CR18] Hébuterne X, Lemarié E, Michallet M, Montreuil CB, Schneider SM, Goldwasser F (2014). Prevalence of malnutrition and current use of nutrition support in patients with cancer. J Parenter Enteral Nutr..

[CR19] Heidegger CP, Berger MM, Graf S, Zingg W, Darmon P, Constanza MC (2013). Optimisation of energy provision with supplemental parenteral nutrition in critically ill patients: a randomised controlled clinical trial. Lancet..

[CR20] Herbert G (2018). Early enteral nutrition within 24 hours of lower gastrointestinal surgery versus later commencement for length of hospital stay and postoperative complications. Cochrane Database Syst Rev..

[CR21] Herbert G (2019). Early enteral nutrition within 24 hours of lower gastrointestinal surgery versus later commencement for length of hospital stay and postoperative complications. Cochrane Database Syst Rev.

[CR22] Heyland DK, Dhaliwal R, Jiang X, Day AG (2011). Identifying critically ill patients who benefit the most from nutrition therapy: the development and initial validation of a novel risk assessment tool. Crit Care..

[CR23] Instituto Nacional de Câncer José Alencar Gomes da Silva (2016). Consenso nacional de nutrição oncológica: volume II.

[CR24] Khalid I, Doshi P, DiGiovine B (2010). Early enteral nutrition and outcomes of critically ill patients treated with vasopressors and mechanical ventilation. Am J Crit Care..

[CR25] Lambert E, Carey S (2016). Practice guideline recommendations on perioperative fasting: a systematic review. J Parenter Enteral Nutr..

[CR26] LeGall JR, Loirat P, Alpérovitch A (1986). APACHE II: a severity of disease classification system. Crit Care Med..

[CR27] Ljungqvist O, Scott M, Fearon KC (2017). Enhanced recovery after surgery: a review. JAMA Surg..

[CR28] Maltby JR (2006). Fasting from midnight: the history behind the dogma. Best Pract Res Clin Anaesthesiol..

[CR29] McClave SA, Taylor BE, Martindale RG (2016). Guidelines for the Provision and assessment of nutrition support therapy in the adult critically Ill patient: society of critical care medicine (SCCM) and american society for parenteral and enteral nutrition (A.S.P.E.N.). J Parenter Enteral Nutr..

[CR30] Mitchell CO, Lipschitz DA (1982). Arm length measurement as an alternative to height in nutritional assessment of the elderly. J Parenter Enteral Nutr..

[CR31] Nelson G, Bakkum-Gamez J, Kalogera E, Glaser G, Altman A, Meyer LA (2019). Guidelines for perioperative care in gynecologic/oncology: Enhanced Recovery After Surgery (ERAS) Society recommendations-2019 update. Int J Gynecol Cancer..

[CR32] Osland E, Yunus RM, Khan S, Memon MA (2011). Early versus traditional postoperative feeding in patients undergoing resectional gastrointestinal surgery: a meta-analysis. J Parenter Enteral Nutr..

[CR33] Reignier J, Boisramé-Helms J, Brisard L, Lascarrou JB, Ait Hssain A, Anguel N (2018). Enteral versus parenteral early nutrition in ventilated adults with shock: a randomised, controlled, multicentre, open-label, parallel-group study (NUTRIREA-2). Lancet..

[CR34] Sandrucci C, Beets G, Braga M, Dejong K, Demartines N (2018). Perioperative nutrition and enhanced recovery after surgery in GI cancer patients. A position paper by the ESSO task force in collaboration with the ERAS society (ERAS coalition). Eur J Surg Oncol..

[CR35] Shida D, Tagawa K, Inada K (2017). Modified enhanced recovery after surgery (ERAS) protocols for patients with obstructive colorectal cancer. BMC Surg..

[CR36] Silva Fink J (2015). Daniel de Mello P, Daniel de Mello E. Subjective global assessment of nutritional status – a systematic review of the literature. Clin Nutr..

[CR37] Silva JM, Malboulsson LMS, Nuevo HL (2010). Aplicabilidade do escore fisiológico agudo simplificado (SAPS 3) em hospitais brasileiros. Rev Bras Anestesiol..

[CR38] Silva FR, Oliveira MG, Souza AS, Figueroa JN, Santos CS (2015). Factors associated with malnutrition in hospitalized cancer patients: a croos-sectional study. Nutr J..

[CR39] Sogayar AM, Machado FR, Rea-Neto A, Dornas A, Grion CM, Lobo SM (2008). A multicentre, prospective study to evaluate costs of septic patients in Brazilian intensive care units. Pharmacoeconomics..

[CR40] Vallejo KP, Martínez CM, Matos Adames AA, Fuchs-Tarlovsky V, Nogales GCC, Paz RER (2017). Current clinical nutrition practices in critically ill patients in Latin America: a multinational observational study. Crit Care..

[CR41] van Zelm R, Coeckeberghs E, Semeus W, Wolthuis A, Bruyneel L, Panella M, Vanhaecht K (2020). Effects of implementing a care pathway for colorectal cancer surgery in ten European hospitals: an international multicenter pre–post-test study. Updates Surg..

[CR42] Vincent JL, Moreno R, Takala J (1996). The SOFA (Sepsis-related Organ Failure Assessment) score to describe organ dysfunction/failure: on behalf on the working group on sepsis-related problems of the european society of intensive care medicine. Intensive Care Med..

[CR43] Vonlanthen R, Slankamenac K, Breitenstein S, Puhan MA, Muller MK, Hahnloser D (2011). The impact of complications on costs of major surgical procedures: a cost analysis of 1200 patients. Ann Surg..

[CR44] Weimann A, Braga M, Carli F (2017). ESPEN guideline: clinical nutrition in surgery. Clin Nutr..

[CR45] Williams DGA, Molinger J, Wischmeyer PE (2019). The malnourished surgery patient: a silent epidemic in perioperative outcomes?. Curr Opin Anaesthesiol..

[CR46] Wischmeyer PE, Carli F, Evans DC (2018). American society for enhanced recovery and perioperative quality initiative joint consensus statement on nutrition screening and therapy within a surgical enhanced recovery pathway. Anesth Analg..

[CR47] Zampieri FG, Soares M, Borges LP, Salluh JIF, Ranzani OT (2017). The epimed monitor ICU Database®: a cloud-based national registry for adult ICU patients in Brazil. Rev Bras Ter Intensiva..

[CR48] Zusman O, Theilla M, Cohen J, Kagan I, Bendavid I, Singer P (2016). Resting energy expenditure, calorie and protein consumption in critically ill patients: a retrospective cohort study. Crit Care..

