# Supplementary material for: Postoperative fasting is associated with longer ICU stay in oncologic patients undergoing elective surgery
Source: Perioper Med (Lond). 2022 Aug 2;11:29. doi: 10.1186/s13741-022-00261-4 (PMC9344771; doi:10.1186/s13741-022-00261-4)
Supplement: Supplementary file 1 — Additional file 1: Supplemental Table 3. Univariate analysis of the outcomes of interest of the patients included in the study. Supplemental Table 4. Glucose blood level and use of insulin during the first days of the study. [file 13741_2022_261_MOESM1_ESM.docx]

**Supplemental Material**

**Supplemental Table 3. Univariate analysis of the outcomes of interest of the patients included in the study**

|  | Overall  n = 109 | Fasting ≤ 24 h  n = 43 | Fasting > 24 h  n = 66 | *P*-value |
| --- | --- | --- | --- | --- |
| Primary outcome | | | | |
| LOS* hospital (days; range) | 25 (15.5–47.5) | 24 (13–41) | 25.5 (17–57) | 0.244 |
| Secondary Outcome | | | | |
| LOS* ICU* (days; range) | 4 (3–7) | 3 (2–5) | 5.5 (4–8.25) | 0.000 |
| 28-day mortality (n; %) | 8.4 (9) | 4.8 (2) | 10.8 (7) | 0.478 |
| Infection (n; %) | 27.5 (30) | 16.3 (7) | 34.8 (23) | 0.057 |

Abbreviations: LOS, length of stay; ICU, intensive care unit

**Supplemental Table 4. Glucose blood level and use of insulin during the first days of the study**

|  | Fasting < 24 h  n = 43 | Fasting > 24 h  n = 66 | *P*-value |
| --- | --- | --- | --- |
|  | | | |
| HGL 1° day (mg; range) | 167 (140–204) | 140 (128–180) | 0.006 |
| LGL 1° day (mg; range) | 120 (105–139) | 114 (98–129) | 0.114 |
| Administration of Insulin 1° day (%) | 32.6 (14) | 19.7 (13) | 0.099 |
| HGL 2° day (mg; range) | 150 (120–181) | 142 (122–166) | 0.455 |
| LGL 2° day (mg; range) | 110 (96–137) | 110 (87–127) | 0.445 |
| Administration of Insulin 2° day (%) | 20.9 (9) | 14.3 (9) | 0.269 |
| HGL 3° day (mg; range) | 137 (114–150) | 145 (123–170) | 0.373 |
| LGL 3° day (mg; range) | 103 (96–121) | 112 (89–131) | 0.427 |
| Administration of Insulin 3° day (%) | 14.3 (6) | 15.1 (10) | 0.585 |

Abbreviations: HGL, high blood glucose level; LGL, low blood glucose level
